# *Myt3* suppression sensitizes islet cells to high glucose-induced cell death via Bim induction

**DOI:** 10.1038/cddis.2016.141

**Published:** 2016-05-19

**Authors:** B R Tennant, B Vanderkruk, J Dhillon, D Dai, C B Verchere, B G Hoffman

**Affiliations:** 1 Child and Family Research Institute, British Columbia Children's Hospital, 950 W28th Avenue, Vancouver, British Columbia, Canada V5Z 4H4; 2 Department of Surgery, University of British Columbia, Vancouver, British Columbia, Canada V5Z 4E3; 3 Department of Pathology and Laboratory Medicine, University of British Columbia, Vancouver, British Columbia, Canada V6T 2B5

## Abstract

Diabetes is a chronic disease that results from the body's inability to properly control circulating blood glucose levels. The loss of glucose homoeostasis can arise from a loss of *β*-cell mass because of immune-cell-mediated attack, as in type 1 diabetes, and/or from dysfunction of individual *β*-cells (in conjunction with target organ insulin resistance), as in type 2 diabetes. A better understanding of the transcriptional pathways regulating islet-cell survival is of great importance for the development of therapeutic strategies that target *β*-cells for diabetes. To this end, we previously identified the transcription factor *Myt3* as a pro-survival factor in islets following acute suppression of *Myt3 in vitro*. To determine the effects of *Myt3* suppression on islet-cell survival *in vivo*, we used an adenovirus to express an shRNA targeting *Myt3* in syngeneic optimal and marginal mass islet transplants, and demonstrate that suppression of *Myt3* impairs the function of marginal mass grafts. Analysis of grafts 5 weeks post-transplant revealed that grafts transduced with the sh*Myt3* adenovirus contained ~20% the number of transduced cells as grafts transduced with a control adenovirus. In fact, increased apoptosis and significant cell loss in the sh*Myt3*-transduced grafts was evident after only 5 days, suggesting that *Myt3* suppression sensitizes islet cells to stresses present in the early post-transplant period. Specifically, we find that *Myt3* suppression sensitizes islet cells to high glucose-induced cell death via upregulation of the pro-apoptotic Bcl2 family member Bim. Taken together these data suggest that Myt3 may be an important link between glucotoxic and immune signalling pathways.

Type 1 diabetes (T1D) results from autoimmunity gradually leading to a loss of *β*-cells and hyperglycaemia.^1^ It is thought that recruitment and activation of auto-reactive T cells targeting *β*-cells is the primary inflammatory process driving the disease.^2, 3, 4^ Recruited immune cells produce Fas, perforin, IL-1*β*, TNF*α* and IFN*γ* that bind to receptors on the surface of *β*-cells leading to activation of downstream signalling cascades and inducing changes in *β*-cell gene expression resulting in the induction of *β*-cell dysfunction and apoptosis.^5, 6, 7, 8, 9, 10^ It is essential that we develop strategies to halt the immune assault on the islets, and prevent cytokine-induced changes in *β*-cell gene expression and apoptosis. There remain, however, significant gaps in our understanding of the regulatory processes and downstream mediators of cytokine-induced *β*-cell dysfunction and death that need to be addressed in order to develop novel therapeutic approaches to block the detrimental effects of immune assault during diabetes progression and islet transplantation.

To improve our understanding of cytokine-induced gene expression changes, we chose to focus on myelin transcription factor 3 (*Myt3*), also known as suppressor of tumorigenicity 18 (*St18*), a C2HC-type zinc-finger transcription factor that is highly expressed in pancreatic islets.^11^ We previously established that exposure of islets to IL-1*β*, TNF*α* and IFN*γ* suppresses *Myt3* expression in a concentration- and time-dependent manner. We further demonstrated that suppression of *Myt3* in islets *ex vivo*, for as little as 48 h, results in increased islet-cell death.^12^ These data suggested that *Myt3* may participate in the development of diabetes downstream of immune assault. Here, to determine the role of *Myt3* in islet function and survival *in vivo* we performed optimal and marginal mass syngeneic islet transplants and assessed glucose homeostasis and graft histology. We hypothesized that in this model *Myt3* suppression would induce islet-cell apoptosis, supporting our hypothesis that *Myt3* is a key regulator of islet-cell survival.

## Results

### *Myt3* suppression impairs marginal but not optimal syngeneic islet graft function

**Figure 1:**
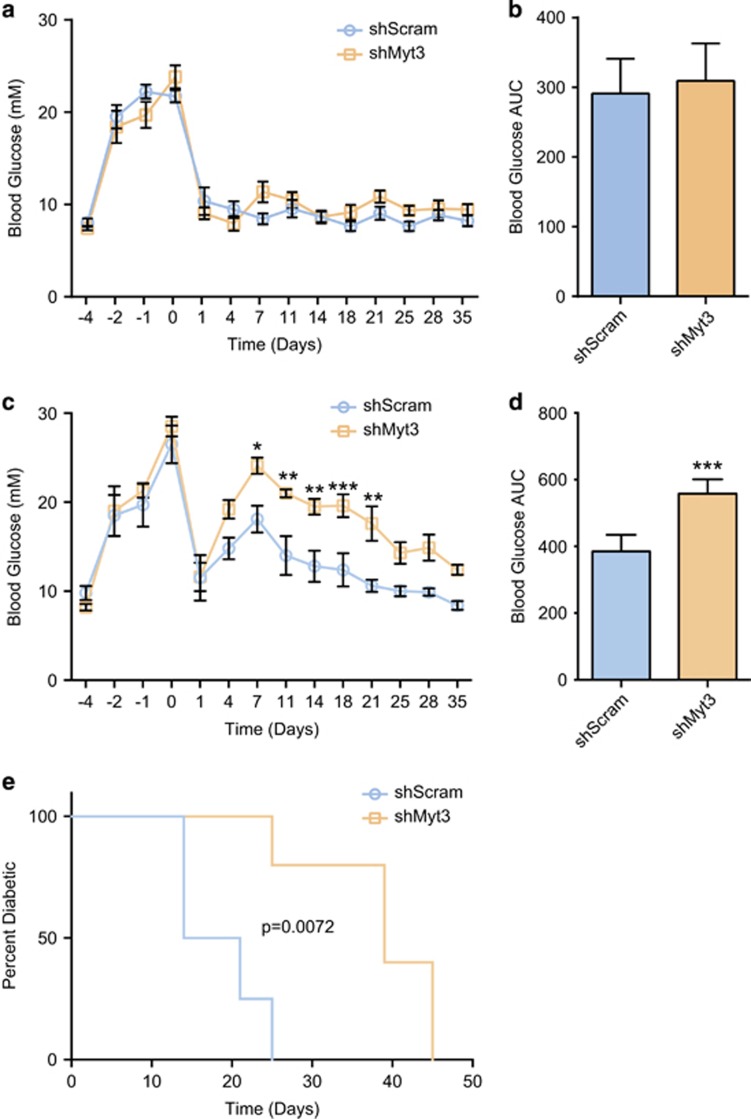
*Myt3* suppression impairs marginal but not optimal islet graft function. (**a**) Random-fed blood glucose measurements for mice transplanted with an optimal mass (300 islets) of sh*Scramble*- or sh*Myt3*-transduced islets. Results are represented as mean±S.E.M. of seven independent experiments. (**b**) Area under the curve for blood glucose measurements from day 1 to 35 post-optimal transplant. Results are represented as mean±S.E.M. of seven independent experiments. (**c**) Random-fed blood glucose measurements for mice transplanted with a marginal mass (150 islets) of sh*Scramble*- or sh*Myt3*-transduced islets. Results are represented as mean±S.E.M. of five independent experiments. *P<0.05, **P<0.01, ***P<0.001 comparing mice receiving grafts using sh*Myt3*-transduced islets with mice receiving grafts using sh*Scramble*- transduced islets (two-way ANOVA). (**d**) Area under the curve for blood glucose measurements from day 1 to 35 post-suboptimal transplant. Results are represented as mean±S.E.M. of five independent experiments. ***P<0.001 comparing mice receiving grafts using sh*Myt3*-transduced islets to mice receiving grafts using sh*Scramble*- transduced islets (unpaired *t*-test). (**e**) Kaplan–Meier survival curve comparing time to return to normoglycaemia for mice receiving marginal mass transplants of sh*Scramble*- or sh*Myt3*-transduced islets. Mice were considered non-diabetic after two consecutive blood glucose measurements <12 mM

To confirm the utility of syngeneic islet grafts as a model with which to study the *in vivo* effects of *Myt3* suppression, we first rendered female C57/B6N mice diabetic via treatment with streptozotocin (STZ) and subsequently transplanted them with an optimal (300) mass of islets and assessed Myt3 expression in the grafts over 5 weeks. Myt3 expression during this time frame appeared to be maintained in the grafts at a similar level as in adult islets, indicating this is a suitable model for studying the effects of *Myt3* suppression on graft survival and function (Supplementary Figure S1). As such, we transplanted STZ-diabetic mice, as above, with either an optimal (300) or a marginal (150) mass of islets transduced with adenoviruses expressing an shRNA targeting *Myt3* (sh*Myt3*) or a scramble control (sh*Scramble*). Monitoring of random-fed blood glucose levels of mice receiving optimal grafts showed that both sh*Scramble* and sh*Myt3*-transduced grafts were equally capable of normalizing blood glucose levels (Figures 1a and b). Further, there was no difference in the ability of sh*Scramble* or sh*Myt3* grafts to respond to a glucose challenge as determined by performing intraperitoneal glucose tolerance tests (IPGTT) 5 days or 5 weeks post-transplant (Supplementary Figures S2a–f).

On the other hand, mice transplanted with a marginal mass of sh*Myt3*-transduced islets had significantly higher blood glucose levels from days 7 to 21 post-transplant (*P*<0.05), as compared with mice transplanted with sh*Scramble*-transduced islets (Figures 1c and d). After day 21, the mice transplanted with sh*Myt3*-transduced islets started to normalize, but still tended to have higher blood glucose levels, and had a significantly delayed time to reestablish normoglycaemia (*P*<0.01; two blood glucose measurements below 12 mM) (Figure 1e). At 5 weeks post-transplant, mice receiving sh*Myt3-*transduced islets were also moderately less able to respond to a glucose challenge, but neither sh*Scramble* and sh*Myt3* grafts were able to re-stabilize blood glucose levels in the time frame of the IPGTT, and this difference was not significant (Supplementary Figures S2g–i). Taken together, these data suggest that, although in mice transplanted with an optimal mass of sh*Myt3*-transduced islets sufficient *β*-cell mass remains to normalize blood levels, in marginal mass transplants *Myt3* suppression impairs the ability of the grafts to establish normoglycaemia.

### *Myt3* suppression increases cell death in syngeneic islet transplants

**Figure 2:**
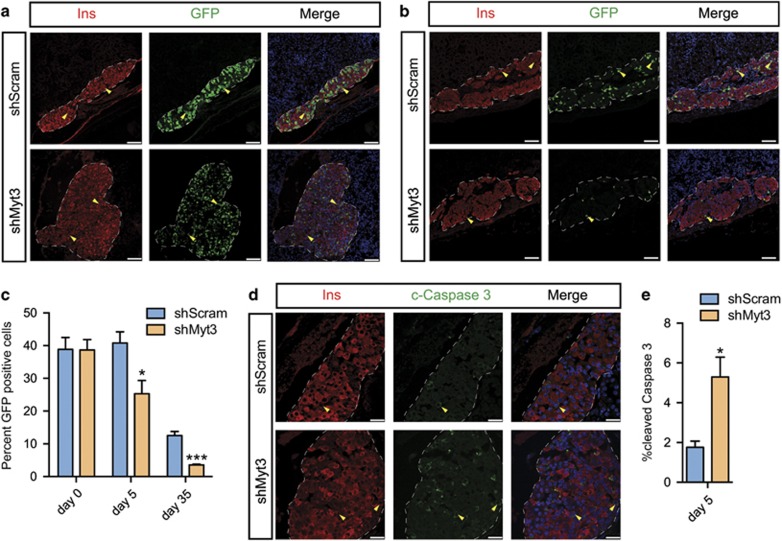
Loss of sh*Myt3*-transduced cells occurs within 5 days of transplantation. sh*Scramble*- and sh*Myt3*-transduced islet grafts (**a**) 5 days or (**b**) 5 weeks post-transplant were stained for insulin (red) and GFP (green) to mark transduced cells. Nuclei are labelled with Topro3 (blue). White dashed line represents the graft boundary. Yellow arrow heads indicate representative insulin and GFP co-positive cells. Scale bars: white bar=75μm. (**c**) Quantification of GFP area in sh*Scramble*- and sh*Myt3*-transduced islet grafts pre-transplant, 5 days and 5 weeks post-transplant. Results are represented as mean±S.E.M. of three independent experiments. *P<0.05, ***P<0.001 comparing grafts using sh*Myt3*-transduced islets *versus* grafts using sh*Scramble*-transduced islets (unpaired *t*-test). (**d**) Grafts were stained with insulin (red) and cleaved caspase-3 (green). Nuclei are labelled with Topro3 (blue). White dashed line represents the graft boundary. Yellow arrow heads indicate representative insulin and cleaved caspase-3 co-positive cells. Scale bars: white bar=25 *μ*m. (**e**) Quantification of the percent of graft cells that stained for cleaved caspase-3. Results are represented as mean±S.E.M. of three independent experiments. *P<0.05 comparing grafts using sh*Myt3*-transduced islets *versus* grafts using sh*Scramble*-transduced islets (unpaired *t*-test)

To determine whether *Myt3* suppression induced cell loss in the islet grafts, we performed immunohistochemistry on grafts harvested from optimal islet mass transplants, to focus on the direct effects of *Myt3* on *β*-cell loss, in the absence of ER stress and other confounding factors that might occur in marginal mass transplants. FACS analysis of transduced islets prior to transplant, based on GFP (which is co-expressed with the shRNA), showed a transduction efficiency of 35–40% for both sh*Myt3* and sh*Scramble* adenoviruses (Supplementary Figures S3a and b). Analysis of grafts 5 days post-transplant showed that sh*Scramble*-transduced grafts still contained approximately 40% transduced cells, while sh*Myt3*-transduced grafts contained only ~25% transduced cells (*P*<0.05) (Figures 2a and c). In contrast, culture of sh*Scramble*- or sh*Myt3*-transduced islets *in vitro* for 5 days on the extracellular matrix 804G did not affect the number of GFP-positive islet cells (Supplementary Figures S3c and d), or significantly increase levels of apoptosis (Supplementary Figures S3e and f). Meanwhile, quantification of GFP area in the grafts 5 weeks post-transplant showed that sh*Myt3* grafts contained only 2–3% GFP-positive cells, five times less than the number found in sh*Scramble* grafts (13% *P*<0.001) (Figures 2b and c). Given that the cell loss seemed to be most rapid early in the engraftment period (nearly 40% of sh*Myt3*-transduced cells were lost in the first 5 days after transplant), we assessed the number of cleaved caspase-3-positive cells 5 days post-transplant. At this time, sh*Scramble*-transduced grafts contained only a few cleaved caspase-3-positive cells (~1.7%), while sh*Myt3*-transduced grafts contained three times as many cleaved caspase-3-positive cells (~5.3%) (Figures 2d and e), indicating that *Myt3* suppression significantly increased the level of apoptosis in the grafts at this time (*P*<0.05).

**Figure 3:**
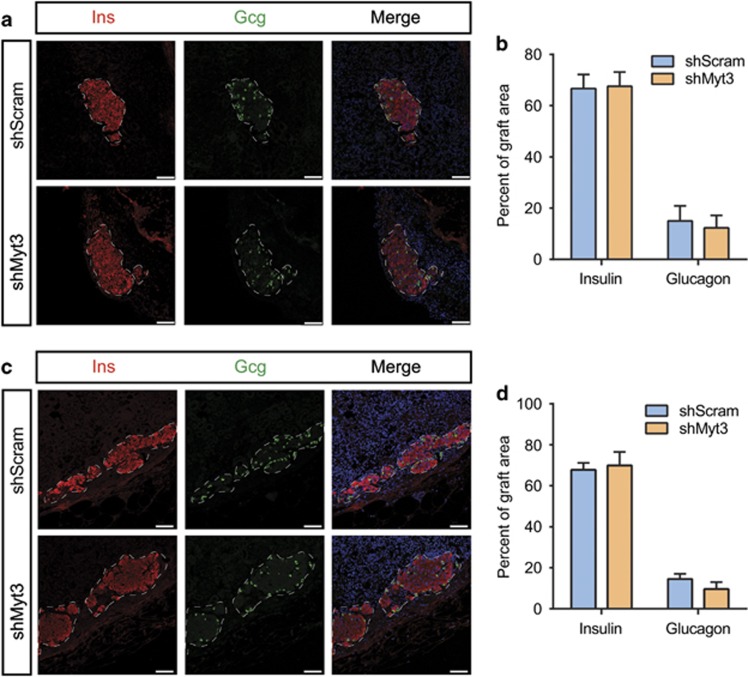
sh*Myt3*-transduced cells are lost from grafts without altering graft composition. (**a**) sh*Scramble*- and sh*Myt3*-transduced islet grafts 5 days post-transplant were stained for insulin (red) and glucagon (green) to mark transduced cells. Nuclei are labelled with Topro3 (blue). White dashed lines represent the graft boundaries. Scale bars: white bar=75 *μ*m. (**b**) Quantification of insulin and glucagon area in (**a**). Results are represented as mean±S.E.M. of three independent experiments. (**c**) sh*Scramble*- and sh*Myt3*-transduced islet grafts 5 weeks post-transplant were stained with insulin (red) and glucagon (green). Nuclei are labelled with Topro3 (blue). White dashed lines represent the graft boundaries. Scale bars: white bar=75 *μ*m. (**d**) Quantification of insulin and glucagon area in (**c**). Results are represented as mean±S.E.M. of three independent experiments

The 35–40% islet-cell transduction efficiency we achieved likely explains why no observable graft dysfunction was detectable in mice receiving an optimal mass of sh*Myt3-*transduced islets. Indeed, quantification of the number of *α*- and *β*-cells in these grafts at both 5 days (Figures 3a and b) and 5 weeks (Figures 3c and d) post-transplant indicated that adequate numbers of *α*- and *β*-cells remain to maintain normoglycaemia despite the selective loss of sh*Myt3*-transduced islet cells. Regardless, the fact that *Myt3* suppression significantly increased the level of apoptosis in sh*Myt3*-transduced grafts after only 5 days, but did not significantly increase islet-cell apoptosis over the same time frame *in vitro*, suggests that *Myt3* suppression sensitizes islet cells to undergo apoptosis in response to stresses faced specifically within the grafts.

### *Myt3* suppression increases chemokine expression but not immune infiltration

Cytokines produced by islet-infiltrating immune cells induce the expression of pro-inflammatory chemokines and cytokines in *β*-cells that act to recruit additional immune cells, contributing to *β*-cell dysfunction and apoptosis.^2, 3, 4, 6, 7, 8, 13, 14, 15, 16, 17, 18^ Reanalysis of our previously generated RNA-seq data^19^ showed that *Myt3* suppression induces the expression of several chemokines, including *Ccl2, Cxcl10* and *Ccl20* (Supplementary Figure S4a), and thus we sought to determine the significance of this increased chemokine expression to sh*Myt3*-transduced islet-cell loss in our grafts. Quantitative real-time polymerase chain reaction (qPCR) validation demonstrated that a 2.4-fold (*P*<0.001) reduction in *Myt3* expression resulted in a significant increase in the expression of *Ccl2* (3.7-fold, *P*⩽0.05)*, Cxcl10* (7.7-fold, *P*⩽0.05) and *Ccl20* (8.3-fold, *P*⩽0.05) (Supplementary Figure S4b). To determine whether sh*Myt3-*induced pro-inflammatory chemokine expression was sufficient to drive recruitment of immune cells, islet grafts were immunostained for the pan-immune cell marker CD45 at both 5 days and 5 weeks post-transplant. However, quantification of immune cell infiltration in the grafts showed that infiltration was negligible and equivalent between sh*Scramble* and sh*Myt3*-transduced islets (Supplementary Figure S4c). These data suggest that the level of chemokine production induced by *Myt3* suppression is insufficient to drive additional recruitment of immune cells to syngeneic islet grafts.

### *Myt3* suppression sensitizes islet cells to metabolic stress-induced cell death

**Figure 4:**
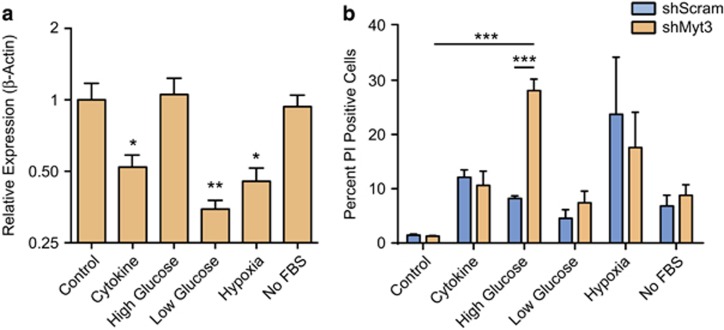
*Myt3* suppression sensitizes islet cells to high glucose-induced cell death. (**a**) *Myt3* expression was determined by qPCR following exposure to either 11 mM glucose (Control), 0.4 ng/ml IFN*γ*, 0.07 ng/ml IL-1*β* and 0.04 ng/ml TNF*α* (Cytokine), high glucose (30 mM glucose), low glucose (2.8 mM glucose), hypoxia (1% O_2_) or serum starvation (No FBS). Results are represented as mean±S.E.M. of four independent experiments. *P<0.05, **P<0.01 comparing islet cells exposed to the indicated treatments as compared with 11 mM glucose (Control) (one-way ANOVA). (**b**) Cell death was measured by propidium iodide incorporation in dispersed islet cells transduced with sh*Scramble* or sh*Myt3* adenoviruses were exposed to either 11 mM glucose (Control), 0.4 ng/ml IFN*γ*, 0.07 ng/ml IL-1*β* and 0.04 ng/ml TNF*α* (Cytokine), high glucose (30 mM glucose), low glucose (2.8 mM glucose), hypoxia (1% O_2_) or serum starvation (No FBS). Results are represented as mean±S.E.M. of five independent experiments. ***P<0.001 comparing the indicated bars (two-way ANOVA)

Given that *Myt3* suppression induced a significant level of islet-cell loss during the time frame in which engraftment is occurring, we next sought to determine whether *Myt3* suppression sensitized islet cells to undergo cell death in response to stresses experienced during this process.^20, 21^ Specifically, during engraftment, islet transplants are exposed to significant metabolic stress, including hypoxia and nutrient deprivation prior to revascularization, hyperglycaemia upon initial revascularization, as well as oxidative stress, and stress induced by exposure to cytokines produced by immune cells recruited to the graft site.^21, 22, 23, 24, 25^ We previously determined that cytokine exposure suppresses *Myt3,*^12^ so we initially sought to determine the effect of these additional cell stresses on *Myt3* expression. For this we exposed islet cells to cytokines (0.4 ng/ml IFN*γ*, 0.07 ng/ml IL-1*β* and 0.04 ng/ml TNF*α*), high glucose (30 mM glucose), low glucose (2.8 mM glucose), hypoxia (1% O_2_) or nutrient deprivation (serum withdrawal). This confirmed that *Myt3* expression is inhibited by cytokine exposure (1.9-fold, *P*<0.01) and demonstrated that hypoxia (2.2-fold, *P*<0.01) and low glucose (2.9-fold, *P*<0.01) similarly inhibit *Myt3* (Figure 4a). To determine whether *Myt3* suppression sensitizes islet cells to these stresses, we transduced dispersed islet cells grown on 804G with our sh*Myt3* and sh*Scramble* adenoviruses and subsequently exposed the cells to each of the cell stressors and monitored cell death (by PI incorporation). Exposure of sh*Myt3*-transduced islet cells to high glucose for 48 h increased the number of PI-positive cells by 3.4-fold (*P*<0.001) as compared with cells transduced with scramble control (Figure 4b), and 22.5-fold (*P*<0.001) as compared with sh*Myt3*-transduced islets in normal glucose conditions. *Myt3* suppression, however, did not significantly affect the level of cell death induced by any of the other stressors. These results indicate that *Myt3* suppression sensitizes islet cells to high glucose-induced cell death.

**Figure 5:**
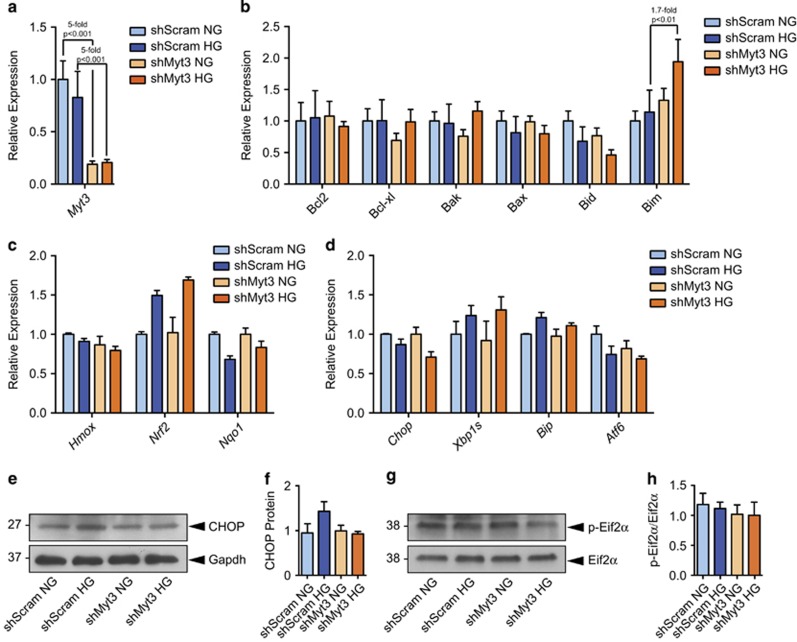
*Myt3* suppression increases high glucose-induced Bim expression in islet cells. qPCR analysis of (**a**) *Myt3* expression, (**b**) pro- and anti-apoptotic Bcl-family genes and (**c**) ER stress or (**d**) oxidative stress-induced genes, in sh*Scramble*- or sh*Myt3*-transduced islets under normal glucose (NG) or high glucose (HG) conditions. (**e**) CHOP and (**g**) phosphorylated eIF2*α* *versus* total eIF2α protein levels were determined by western blot in sh*Scramble*- or sh*Myt3*-transduced islets under NG or HG conditions. (**f**) Quantification of CHOP protein as a fraction of Gapdh protein in (**c**). Results are represented as mean±S.E.M. of three independent experiments. (**h**) Quantification of phosphorylated eIF2*α* as a fraction of total eIF2*α* protein levels in (**g**). Results are represented as mean±S.E.M. of three independent experiments

Since *Myt3* suppression sensitized islet cells specifically to high glucose-induced cell death, we next determined whether *Myt3* suppression exacerbates hyperglycaemia-induced pro-apoptotic, ER- or oxidative-stress-related gene expression. *Myt3* suppression resulted in a 1.7-fold increase (*P*<0.01) in high glucose-induced *Bim* expression as compared with sh*Scramble-*treated controls, but had little effect on the expression of other pro-apoptotic genes, or genes induced by ER- or oxidative stress (Figures 5a–d). Further, levels of C/EBP homologous protein (CHOP), or phosphorylated eukaryotic initiation factor-2 (eIF2*α*), were also unaltered, implying *Myt3* suppression does not affect hyperglycaemia-induced ER stress in this context (Figures 5e–g).

**Figure 6:**
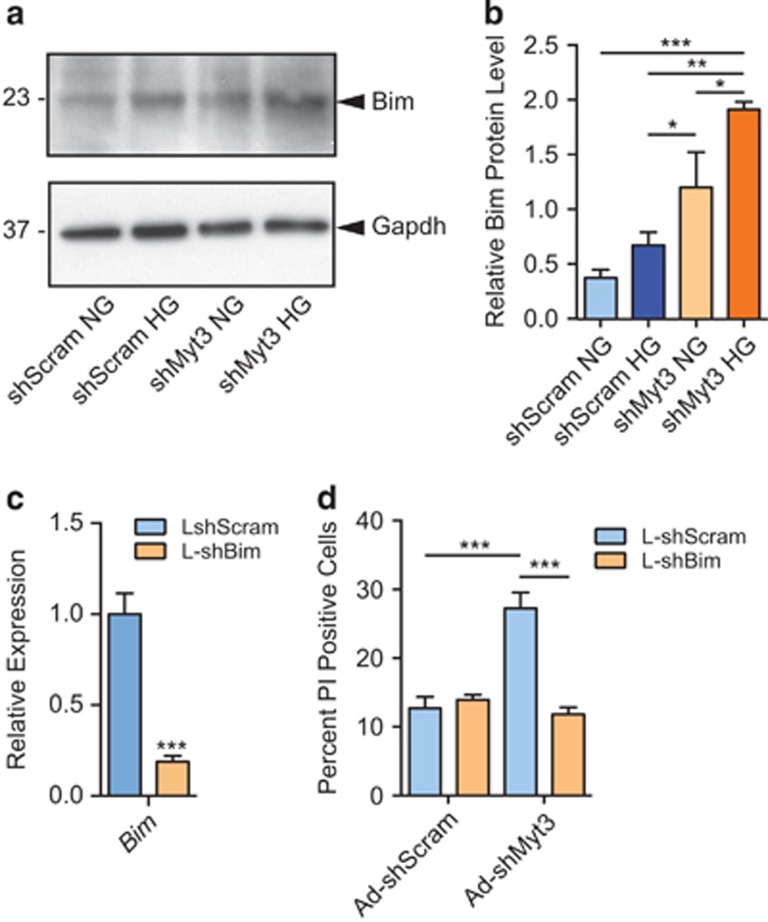
*Myt3* suppression sensitizes islet cells to high glucose-induced cell death via upregulation of Bim. (**a**) Bim protein was determined by western blot in sh*Scramble*- or sh*Myt3*-transduced islets under normal glucose (NG) or high glucose (HG) conditions. (**b**) Quantification of Bim protein as a fraction of Gapdh protein in (**a**). Results are represented as mean±S.E.M. of three independent experiments. *P<0.05, **P<0.01, ***P<0.001 comparing the indicated bars (two-way ANOVA). (**c**) qPCR analysis of *Bim* expression in dispersed islet cells transduced with sh*Scramble* (L-shScram) or sh*Bim* (L-shBim) expressing lentiviruses. Results are represented as mean±S.E.M. of four independent experiments. ***P<0.001 comparing L-shScram to L-shBim transduced cells (unpaired *t*-test). (**d**) Cell death was determined by propidium iodide incorporation in dispersed islet cells co-transduced with lentiviral sh*Scramble* (L-shScram) or sh*Bim* (L-shBim), and adenoviral sh*Scramble* (Ad-shScram) or sh*Myt3* (Ad-shMyt3), under high glucose conditions. Results are represented as mean±S.E.M. of four independent experiments. ***P<0.001 comparing the indicated bars (two-way ANOVA)

These results suggest that *Myt3* suppression may specifically sensitize islet cells to high glucose-induced cell death via Bim. In support of this, *Myt3* suppression resulted in an approximately fivefold increase (*P*<0.001) in Bim protein levels (Figures 6a and b) in cells treated with high glucose. Next, we measured islet-cell death in sh*Myt3*-transduced cells exposed to high glucose and co-transduced with a lentivirus expressing an shRNA targeting *Bim*, which resulted in a five-fold reduction in *Bim* expression (Figure 6c)*. Bim* suppression essentially abrogated the increase in sh*Myt3*-transduced islet-cell apoptosis in response to high glucose, as compared with sh*Scramble-*transduced controls (Figure 6d). These data indicate that exposure of islet cells, in which *Myt3* is suppressed, to high glucose results in Bim upregulation, causing an imbalance in pro-apoptotic and pro-survival Bcl2 family members that results in islet-cell death.

## Discussion

During diabetes progression exposure of *β*-cells to cytokines produced by infiltrating immune cells activates pro-inflammatory signalling cascades that ultimately lead to amplification of inflammatory signals and initiation of apoptosis.^2, 3, 4, 6, 7, 8, 13, 14, 15, 16, 17^ We previously demonstrated that the transcription factor *Myt3* is one of the downstream targets of IL-1*β*, TNF*α* and IFN*γ* signalling in *β*-cells, and that its suppression is sufficient to induce a two-fold increase in apoptosis when islet cells are cultured *in vitro*, without affecting islet function.^12^ However, these analyses were conducted over a very short period (~48 h), in the absence of extracellular matrix, and did not provide insight into the longer-term effects of *Myt3* suppression on islet function and survival *in vivo*. In the current work, we sought to address this deficit using a syngeneic islet transplant model to examine the effects of *Myt3* suppression on graft survival and function.

We demonstrate that, although neither body weight nor blood glucose homoeostasis were significantly affected in mice transplanted with an optimal mass of sh*Myt3-*transduced islets, in marginal mass transplants *Myt3* suppression substantially delays the time required for graft recipients to reestablish normoglycaemia. Further, in optimal mass transplants, by 5 weeks post-transplant only 2–3% of cells remaining in sh*Myt3*-transduced islet grafts were sh*Myt3*-transduced cells. Although, there was also some loss of GFP expression in sh*Scramble*-transduced islet grafts at this time, given that our initial transduction efficiency was ~40%, and the presence of five times fewer GFP-positive cells in the sh*Myt3* grafts than in the sh*Scramble* grafts, this low frequency of sh*Myt3*-transduced cells in the grafts at this time suggests that the majority of sh*Myt3*-transduced cells had undergone cell death. In support of this, *Myt3* suppression significantly increased the level of apoptosis in optimal mass grafts after only 5 days, and the loss of transduced cells was already evident at this time. Importantly, suppression of *Myt3* did not have any significant effect on islet-cell death in islet cells grown *in vitro* on the extracellular matrix 804G for the same amount of time. These data suggest that *Myt3* suppression sensitizes islet cells to undergo apoptosis in response to stresses faced within the islet graft.

*Myt3* suppression in islets *in vitro* leads to the upregulation of several chemokines, likely via *Myt3* suppression indirectly or directly allowing their de-repression, given that islet-cell death was not significantly increased by *Myt3* suppression in these conditions. As the chemokines induced by *Myt3* suppression are capable of recruiting immune cells, we hypothesized their expression might contribute to the loss of islet cells following transplantation.^2, 3, 4, 6, 7, 8, 13, 14, 15, 16, 17^ However, the level of induction of these chemokines appeared to be insufficient to drive the recruitment of additional immune cells to the grafts, suggesting alternate mechanisms are likely responsible for the increased cell death observed in the sh*Myt3*-transduced islet grafts.

Islet transplants are exposed to significant levels of cell stress during the engraftment process.^21^ The increased cell death observed during the first 5 days post-transplant in our model implicated these cell stresses as a potential modifier of sh*Myt3*-induced islet-cell death. In fact, analysis of various cell stressors demonstrated that *Myt3* suppression significantly increased high glucose-induced islet-cell death. In the context of transplantation, this suggests that loss of *Myt3* promotes cell death when the engrafted islets are exposed to high circulating concentrations of blood glucose immediately following transplantation. *Myt3* suppression did not further sensitize islet cells to cytokine-, low glucose- or hypoxia-induced cell death, likely because these stressors already reduce *Myt3* expression. We further show that the sensitivity of islet cells to high glucose-induced cell death following *Myt3* suppression is dependent on the induction of the pro-apoptotic Bcl2 family member Bim. Bim induction is similarly critical to islet-cell death in response to Pdx1 and IRS2 deficiency and in response to glucotoxity.^26, 27, 28^

Exposure of *β*-cells to inflammatory cytokines can exacerbate the effects of glucotoxicity; however, how this ‘glucocytotoxicity' occurs is not well characterized.^29, 30, 31^ Our previous data show that suppression of *Myt3* by inflammatory cytokines plays a significant role in cytokine-induced *β*-cell apoptosis, while our data here indicate that *Myt3* suppression sensitizes *β*-cells to high glucose-induced cell death. Together, these data suggest that Myt3 may act as a mediator of inflammatory and glucotoxic signalling pathway crosstalk, with inflammatory signalling-induced *Myt3* suppression, as may occur in T1D and T2D, sensitizing *β*-cells to undergo cell death in response to hyperglycaemic episodes via allowing precocious Bim activation. We believe that future efforts need to be targeted at uncovering the mechanisms underlying the effects of *Myt3* suppression on *β*-cell apoptosis and determining whether the maintenance of *Myt3* levels can reduce *β*-cell death and dysfunction.

## Materials and Methods

### Mouse maintenance and handling

Mice were maintained according to the guidelines of the Canadian Council on Animal Care using protocols approved by the UBC Animal Care Committee. Mice were housed in a group setting of up to five mice per cage with daily health checks and cage maintenance performed by animal facility staff members. In our colony, mice were maintained in a 12-h light/dark cycle, fed a standard low fat diet (LabDiet, St. Louis, MO, USA) *ad libitum* and their environment was enriched through administration of sunflower seeds and hiding places. Mice were housed in this setting until needed for experiments. C57/B6N (8–12 weeks old) mice were used in all studies.

Mice used in non-survival procedures were anaesthetized using isofluorane inhalation followed by cervical dislocation. Toe pinch reflex was checked prior to cervical dislocation in order to ensure that the animals were at a surgical plane of anaesthesia.

### Islet isolation and culture

Pancreata from 8- to 12-week-old female mice were perfused with 1000 U/ml Collagenase XI (Sigma-Aldrich, Oakville, ON, Canada) and incubated for 15 min at 37 °C. Pancreata were manually disrupted and passed through a 70 μM filter (Corning, New York, NY, USA) and islets were hand-picked. Islets were cultured in RPMI 1640 (11 mM glucose) supplemented with 10% FBS, 50U/ml penicillin/streptomycin and 2 mM l-glutamine at 37° in a 5% CO_2_ humidified incubator. All tissue culture reagents were purchased from ThermoFisher (Burlington, ON, Canada). Islets were transduced with the sh*Scramble* or sh*Myt3* adenoviruses as done previously^12^ and cultured for 48 h prior to transplantation.

### Islet transplantation

Transplant recipient mice were rendered diabetic by injection of 230 mg/kg streptozotocin (Sigma-Aldrich) into the intraperitoneal space. We monitored these animals on a daily basis for signs of illness and measured blood glucose using a One Touch Ultra glucose test meter (LifeScan Canada Ltd, Burnaby, BC, Canada) to determine the induction of hyperglycaemia. Following development of streptozotocin-induced diabetes in these mice, they were transplanted under the kidney capsule with 300 islets for optimal mass transplants or 150 islets for marginal mass transplants. One hour before surgery, mice received subcutaneous injection of a combination of meloxicam (1 mg/kg) and buprenorphine (0.1 mg/kg). The mice were anaesthetized for surgery with isofluorane (2% in 2 l/min oxygen flow), and surgical plane of anasthesia was assessed by toe pinch reflex. A small nick was made in the kidney capsule and the islets were slowly injected below the capsule. The incisions were sutured closed and the mice were allowed to recover from the anaesthetic prior to being returned to their cages.^32^

### Glucose tolerance tests

*In vivo* islet function was assessed by intraperitoneal glucose tolerance test (IPGTT). Mice were fasted for 6 h and injected with 1 g/kg D50 glucose (Sigma-Aldrich), and saphenous blood glucose was measured at 0, 15, 30, 60 and 120 min.

### Immunohistochemistry

Immunohistochemistry was performed on paraffin sections of islets embedded under the kidney capsule. Sections were stained with goat anti-glucagon (1:500; Santa Cruz, Dallas, TX, USA), guinea pig anti-insulin (1:500; Linco, St. Charles, MO, USA), rabbit anti-GFP (1:1000; MBL, Woburn, MA, USA) and rabbit anti-cleaved caspase-3 (1:500; Santa Cruz). Primary antibodies were detected using donkey anti-rabbit Alexa 488 (1:1000; Molecular Probes, Eugene, OR, USA), goat anti-guinea pig Alexa 546 (1:500; Molecular Probes) or donkey anti-goat Alexa 488 (1/1000; Molecular Probes).

### Cell death assay

Islets were isolated as described above and a single-cell suspension was prepared by trypsinization. Two hundred islet equivalents per well were plated in 24-well tissue culture plates treated with 804G, a complete extracellular matrix (ECM) produced by a rat bladder carcinoma cell line.^33^ Cells were transduced with the sh*Scramble* or sh*Myt3* adenoviruses overnight at an MOI of 10 and cultured in the presence or absence of various cell stressors for a further 48 h. RPMI media supplemented with 11 mM glucose (normal glucose) served as the control condition, while stressors included a cytokine cocktail (0.4 ng/ml IFN*γ*, 0.07 ng/ml IL-1*β* and 0.04 ng/ml TNF*α*), serum starvation, low glucose (2.8 mM glucose), high glucose (30 mM glucose) or hypoxia (1% O_2_). The pLKO-Bim shRNA (TRCN0000009692-9695), which targets all Bim isoforms,^26^ and lentiviral control (RHS6848) vectors were purchased from GE Dharmacon (Ottawa, ON, Canada). sh*Scramble* and sh*Myt3*-transduced islets were co-transduced with 2.5 *μ*l lentiviral sh*Scramble* or sh*Bim* (1 × 10^9^ pfu) overnight. The media was changed to fresh 30 mM glucose RPMI and cells were cultured for a further 48 h. We added 1 *μ*l propidium iodide (PI, 1 mg/ml; Sigma-Aldrich) and 0.5 *μ*l Hoescht to each well 24 h prior to imaging. Cells were imaged with an SP8 confocal microscope (Leica, Wetzlar, Germany) and quantification was performed with CellProfiler image analysis software (Broad Institute, Cambridge, MA, USA).

### qPCR

Dispersed islets were either transduced as above or left untransduced and cultured in RPMI media supplemented with the cell stressors described above for 36 h. RNA was isolated using Trizol and the PureLink RNA purification kit (Ambion, Burlington, ON, Canada). cDNA was generated using Superscript III (Invitrogen, Burlington, ON, Canada). Taqman probes were used to quantify *β-actin* and *Myt3,* all other primers were designed using Primer3plus and ordered from IDT. A Viia7 real-time PCR system and SYBR Green supermix (Applied Biosystems, Burlington, ON, Canada) or Taqman Fast Advanced Master Mix (Applied Biosystems) was used for all reactions. cDNA (10ng) was used in each reaction, with all reactions done in triplicate. *β-Actin* was used as an internal control and the change in expression was calculated using 2^−ΔΔCt^.

### Western blot analysis

Cell lysates were prepared from transduced, dispersed islets cultured under normal glucose (11 mM glucose) or high glucose (30 mM glucose) conditions for 48 h. Cells were recovered by trypsinization and lysed by sonication in RIPA buffer (ThermoFisher). Twenty-five micrograms total protein was loaded in each well of a 4–15% TGX gel (Biorad, Mississauga, ON, Canada). Gels were transferred to 0.2 *μ*m polyvinyl difluoride membranes (Biorad) using the Trans-Blot Turbo apparatus (Biorad) according to the manufacturer's instructions. Membranes were probed with antibodies against Bim (1:500; Cell Signaling, Danvers, MA, USA), CHOP (1:500; Santa Cruz), phospho-eIF2α (1:1000; Cell Signaling) and total eIF2α (1:500; Abcam, Cambridge, MA, USA) at 4 °C in 5% skim milk diluted in TBS supplemented with 0.05% Tween-20. Blots were subsequently stripped using the Abcam mild stripping protocol and re-probed with anti-Gapdh (1:10000; Cell Signaling). Donkey anti-Rabbit or anti-Mouse (Santa Cruz) HRP-conjugated secondary antibodies were used at 1:10 000 in PBS for 1 h at room temperature. Membranes were treated with ECL reagent (ThermoFisher) for 1 min prior to developing films.

### Statistical analysis

Statistical analysis was performed using a two-way ANOVA with a Sidak's multiple comparison test for physiological measurements over time. Statistical analysis was performed using an ANOVA with a Tukey's multiple comparison test for qPCR, western blot and cell death measurements. All other data were analysed using an unpaired, two-tailed Student's *t*-test. Data from at least three independent experiments are represented as mean±S.E.M. Statistical significance was accepted at *P*-values <0.05.

## Abbreviations

St18: suppression of tumorigenicity 18
Myt3: myelin transcription factor 3
INF*γ*: interferon-gamma
IL-1*β*: interleukin-1 beta
TNF*α*: tumour necrosis factor-alpha
qPCR: quantitative real-time polymerase chain reaction
MOI: multiplicity of infection
STZ: streptozotocin
IPGTT: intraperitoneal glucose tolerance tests
FACS: fluorescence-activated cell sorting
GFP: green fluorescence protein
TUNEL: terminal deoxynucleotidyl transferase dUTP nick end labelling
shRNA: short hairpin RNA
T1D: type 1 diabetes
T2D: type 2 diabetes
ECM: extracellular matrix
