# Supplementary figures and images for: Myt3 suppression sensitizes islet cells to high glucose-induced cell death via Bim induction
Source: Cell Death Dis. 2016 May 19;7(5):e2233–. doi: 10.1038/cddis.2016.141 (PMC4917670; doi:10.1038/cddis.2016.141)

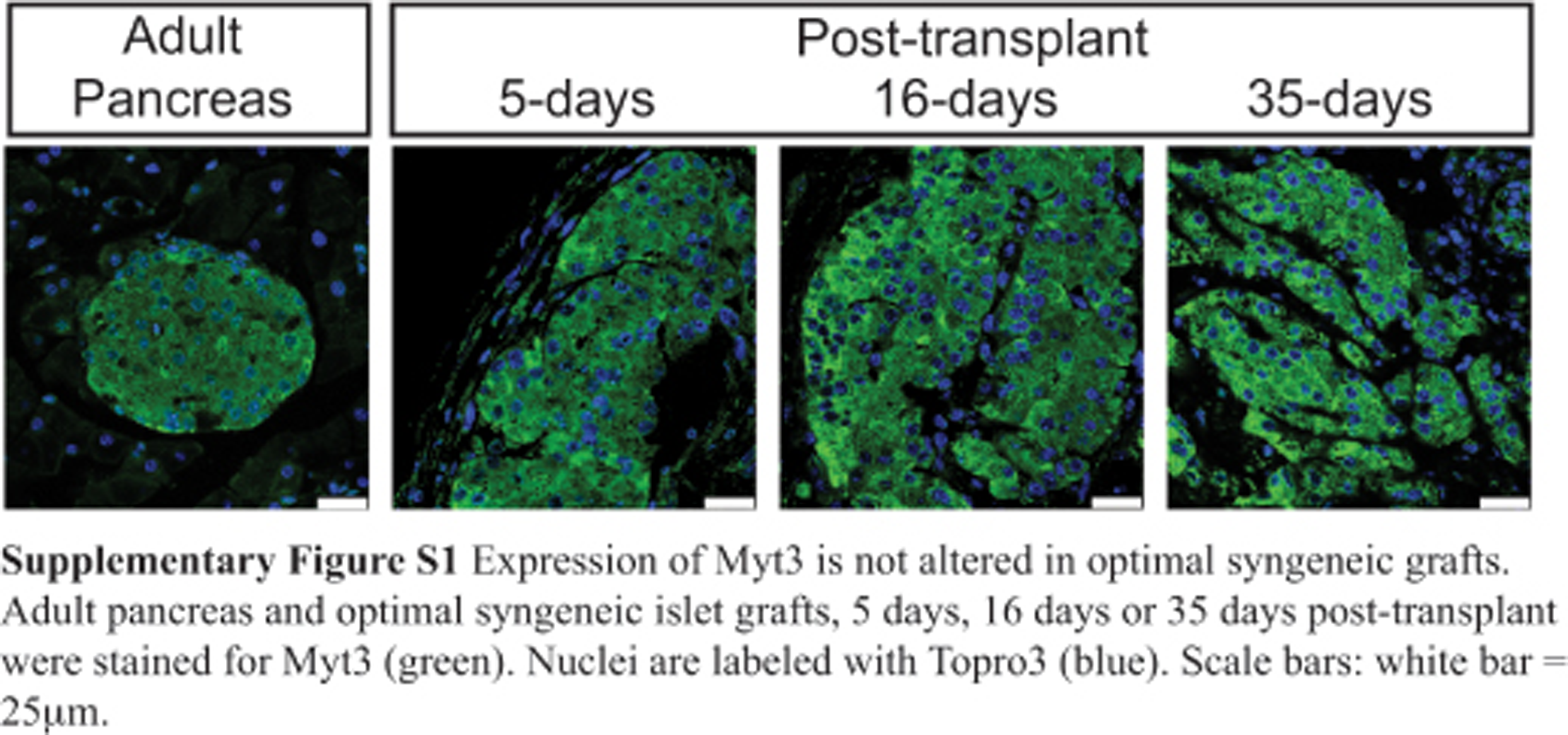

Supplement: Supplementary Figure 1 [file cddis2016141x1.tif]

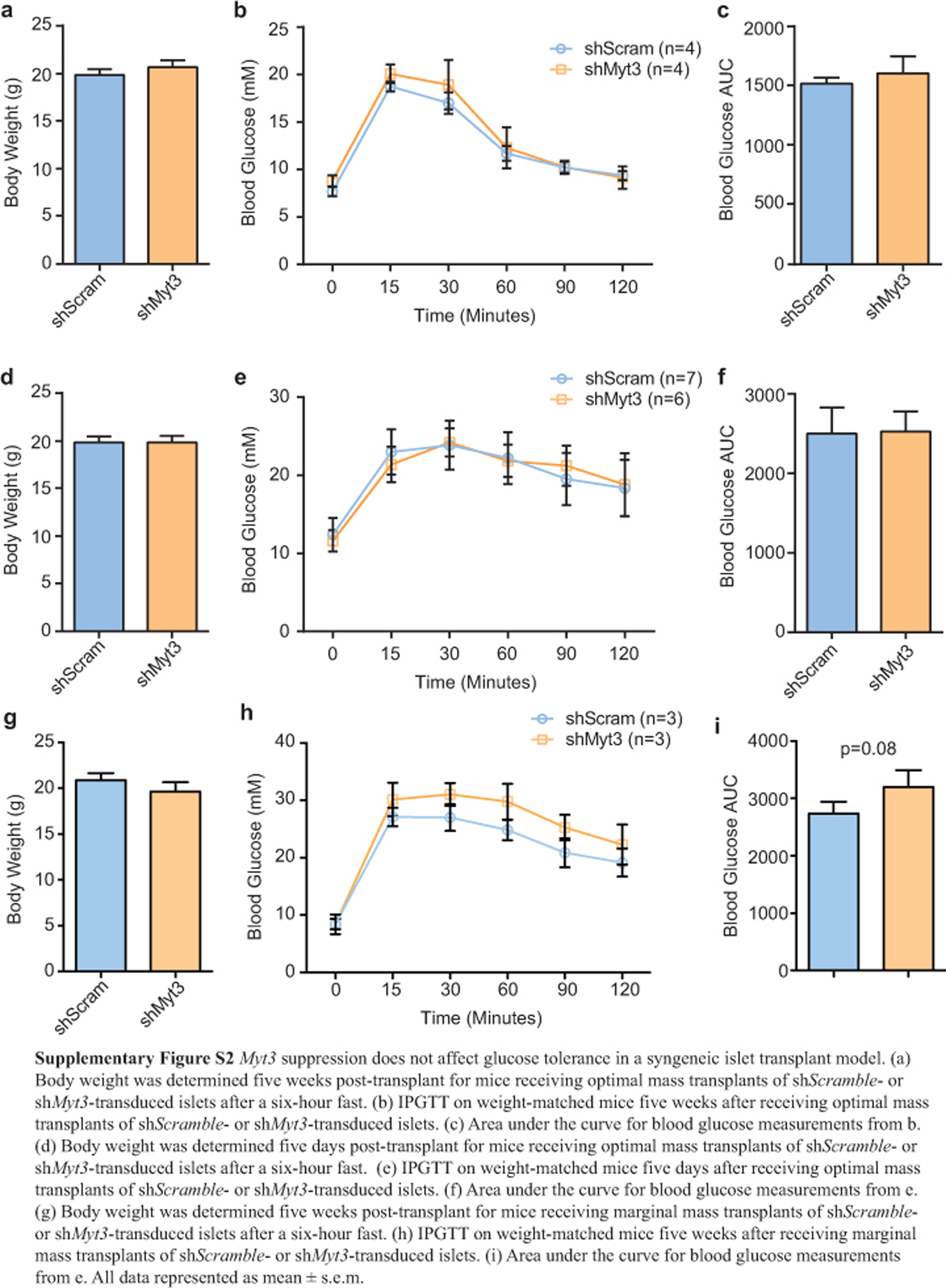

Supplement: Supplementary Figure 2 [file cddis2016141x2.tif]

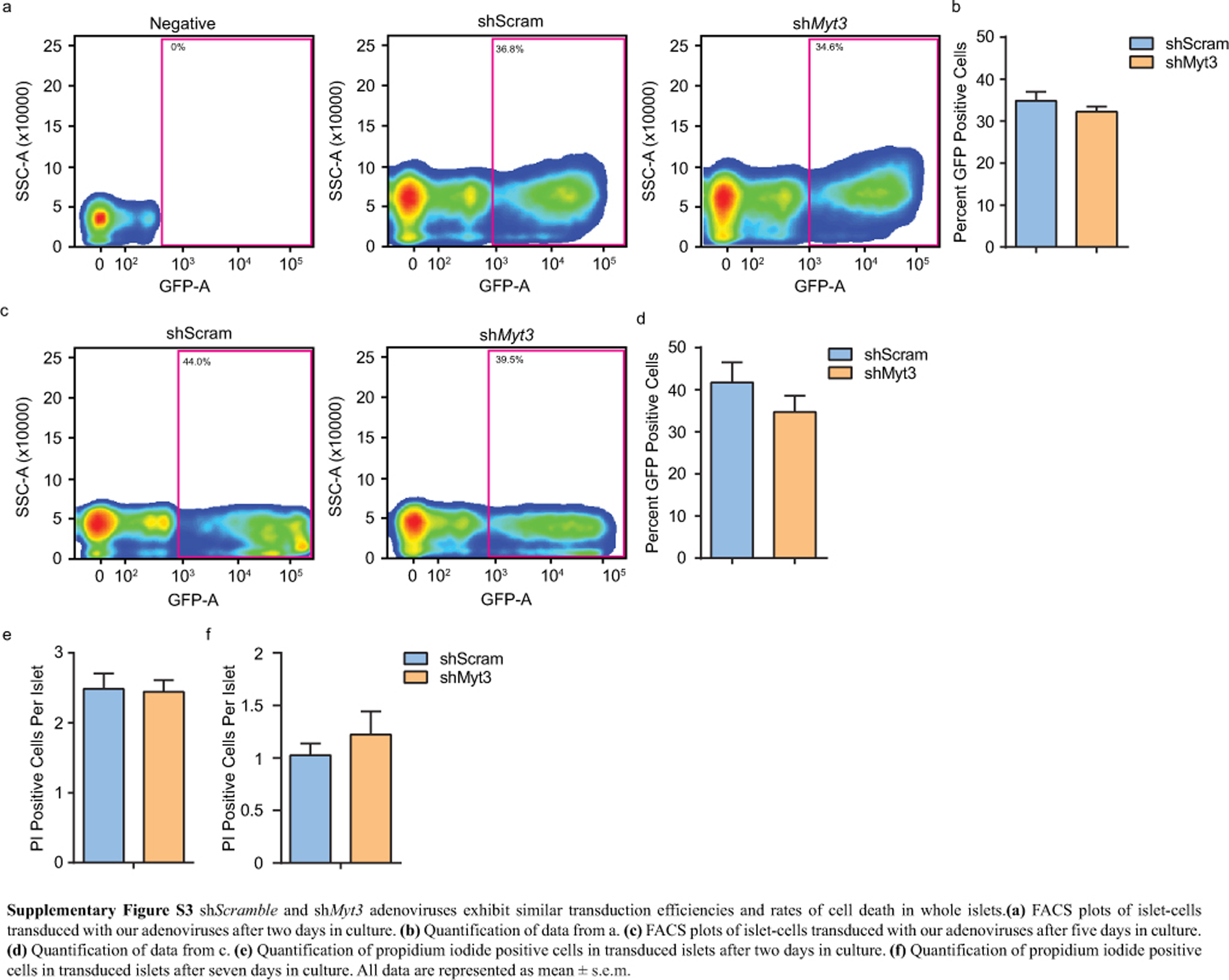

Supplement: Supplementary Figure 3 [file cddis2016141x3.tif]

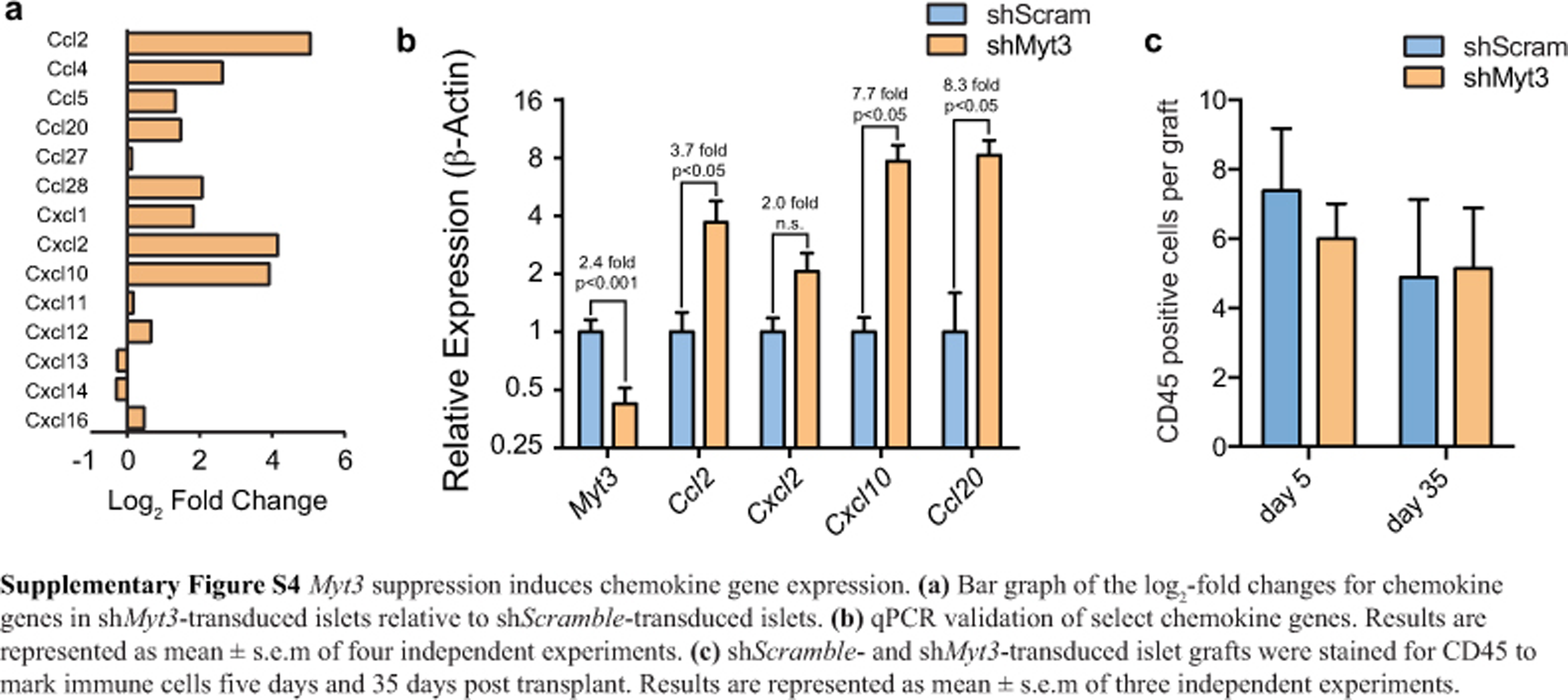

Supplement: Supplementary Figure 4 [file cddis2016141x4.tif]
